# Strength Properties of a Porous Titanium Alloy Ti6Al4V with Diamond Structure Obtained by Laser Power Bed Fusion (LPBF)

**DOI:** 10.3390/ma13225138

**Published:** 2020-11-14

**Authors:** Anna Falkowska, Andrzej Seweryn, Marcin Skrodzki

**Affiliations:** 1 Department of Mechanics and Applied Computer Science, Faculty of Mechanical Engineering, Bialystok University of Technology, 45C Wiejska, 15-351 Bialystok, Poland; a.seweryn@pb.edu.pl; 2 Medgal Orthopaedic Implants & Instruments, 26A Niewodnicka, 16-001 Ksiezyno, Poland; m.skrodzki@medgal.com.pl

**Keywords:** Laser Power Bed Fusion, 3D printing, porosity, Ti6Al4V titanium alloy, diamond structure, mechanical properties

## Abstract

This paper presents the results of experimental research on the strength properties of porous structures with different degrees of density manufactured of Ti6Al4V titanium alloy by Laser Power Bed Fusion. In the experiment, samples with diamond structure of porosity: 34%, 50%, 73% and 81% were used, as well as samples with near-zero porosity. Monotonic tensile tests were carried out to determine the effective values of axial modulus of elasticity, ultimate tensile strength, offset yield strength, ultimate elongation and Poisson ratio for titanium alloys with different porosities. The paper also proposes relationships that can be easily used to estimate the strength and rigidity of a porous material manufactured by 3D printing. They were obtained by the approximation of two quotients. The first one refers to the relationship between the tensile strength of a material with a defined porosity to the strength of full-filled material. The second similarly determines the change in the value of the axial modulus of elasticity. The analysis of microscopic observations of fracture surfaces and also microtomography visualization of the material structure are also presented.

## 1. Introduction

Incremental methods included in rapid prototyping technology allow for the creation of porous structures with mechanical properties (especially elastic parameters) similar to those of bone tissue [1,2]. Moreover, designing and manufacturing the proper structure with a defined porosity can successfully replace damaged bone. Adequate selection of rigidity and strength will allow for better cooperation between the bone and the implant. It can significantly extend the service life of the implant. In the case of an endoprosthesis pin, the external surface is in contact with the bone surface. By introducing porous materials, the modulus of elasticity *E* is reduced, which reduces the contact stresses [3,4,5]. The structures obtained in this way are considered to be one of the most attractive biomaterials for orthopedic implants [6].

In modern implantology, the elements of endoprostheses made of titanium alloy Ti6Al4V are very popular. A well-known manufacturing method is Laser Power Bed Fusion (LPBF), according to ASTM International (American Society for Testing and Materials) [7]. However, this method is identified with definition Selective Laser Melting (SLM). SLM is a commercial name deposited by SLM Solutions Group AG. This additive technique makes it possible to produce a finished element without the need for any additional finishing treatment. Unlike methods where the metal powder is fed through the nozzles and then heated by a point laser beam (such as Laser Engineered Net Shaping—LENS), in the case of the LPBF method, the powder layer is distributed over the entire surface of the element being manufactured. An additional advantage of this method is the possibility to obtain an element with a particular porosity [8,9,10].

The mechanical properties of porous structures, such as stiffness, may be similar to those of bone tissue. It can be obtained using specific values of technological parameters such as laser power, scanning speed and distances between scanning lines or layer thickness. Inadequate selection of the first two parameters, in particular, significantly affects the occurrence of possible defects [9,10,11], causing a decrease in strength parameters of the produced structures. Inappropriately selected laser power and scanning speed results in the supply of an inadequate value of energy density to the sintering powder layer, thus reducing, for example, Young’s modulus or yield strength. In addition, a large amount of voids have a significant impact on the initiation and growth of damage [1,12,13]. The mechanical properties may also be affected by heat treatment of printed elements [5].

To obtain the strength properties of the porous titanium alloy with the diamond structure it is necessary to review research related to this problem. The methodology used in previous monotonic tests of 316L austenitic steel samples [14,15] is very useful and provides a starting point for further research. After fatigue tests two models of fatigue damage accumulation have been developed: strain [16] and stress [17] damage. The presented models enable the estimation of the fatigue life of 316L porous sintered steel obtained by powder metallurgy with a different degree of structure density. 

Most of the strength properties of porous materials of different geometry obtained by the LPBF method [18,19,20] and the degree of structure compaction is determined from the monotonic compression test [21,22,23,24,25,26]. In particular, the strength properties of titanium alloy samples with porosities of 10% to 80% and pore sizes of 600 to 1000 µm were analyzed. Thus, optimal technological parameters producing attractive mechanical properties can be distinguished, e.g., high similarity of Young’s modulus value of porous material to the modulus of bone [21,22,27]. 

The strength properties of porous Ti6Al4V titanium alloy samples printed with the LPBF method, determined on the basis of a monotonic tensile test, are hard to reach in the literature [28,29,30]. More often the values were determined on samples produced by other methods, such as DED (directed energy deposition) or EBM (electron beam melting) [31], or the influence of technological parameters of the LPBF on strength properties [32,33,34] are widely published. Some of the available papers present strength parameters of porous materials obtained by the LPBF method but during compression tests only [35,36,37]. In the case of tensile tests of titanium alloy samples obtained by the LPBF method, the results concerning samples with a structure similar to that of solid material are presented in [38,39], taking into account the orientation of the samples in relation to the printing direction [40,41] or different types of samples obtained by the LPBF method: whole-volume porosity specimens, 1 and 2 mm thick specimens with a solid core and a solid outer surface, 1 mm thick specimens with a porous core and a solid outer surface and solid structure specimens [9]. Strength parameters of titanium alloys are also presented, taking into account the defects formed during the LPBF process [42]. Very interesting results of experimental studies of structures obtained by incremental methods or studies on pantographic structure metamaterials can be found in [43,44]. 

There is a lack of studies on the strength properties of titanium alloys printed with LPBF technology of different structure, obtained by monotonic tensile test. Probably a principal problem in this case is the size of the specimens, which are much larger than those for compression tests, and therefore more expensive to produce. Despite the great interest in the LPBF it is still quite expensive.

This paper presents the results of experimental investigation of strength properties of diamond structure materials made of Ti6Al4V titanium alloy by LPBF. Monotonic tensile tests of samples with porosities: 34%, 50%, 73% and 81% and with near-zero porosity were performed. The effects of porosity on the values of such effective parameters as: axial modulus of elasticity, tensile strength, yield strength and relative elongation is analyzed. To predict the strength and the stiffness of porous material with defined structure simple relations are proposed. The relations are obtained on the basis of approximation of two quotients. The first one refers to the relationship between the tensile strength of a material with a defined porosity to a full-filled material. The second one similarly determines the change in the value of the axial modulus of elasticity. The analysis of microscopy investigation of fractures is also presented.

## 2. Materials and Methods 

The samples for testing were manufactured by LPBF. The building material was Ti6Al4V titanium alloy. It is particularly popular in biomedical applications for elements of joint endoprostheses, stabilizers and dental implants, i.e., elements that replace damaged bone. This is possible mainly due to the high biocompatibility of this alloy [9].

LaserFormTMTi Gr. 23 powder was used to produce samples. The powder was produced by 3DSystems (Los Angeles, CA, USA). The chemical composition of the powder offered by the manufacturer is given in Table 1.

**Figure 1:**
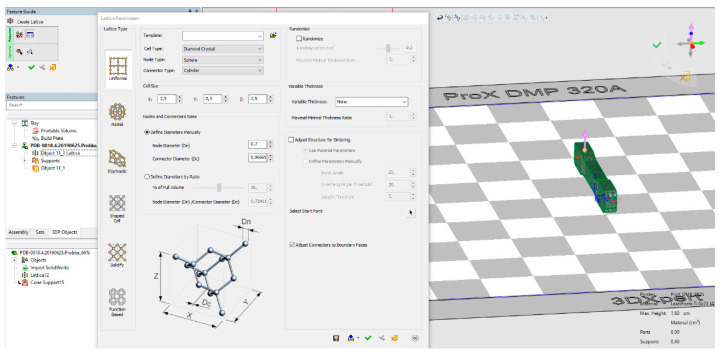
3DXpert interface, where the diamond structure of a sample with different porosities was designed (3DXpert for SolidWorks).

The first step was to create an STL (Standard Triangulation Language) file with the appropriate structure and its density. For this purpose, a 3DXpert 14.0 SP3 program was used (Figure 1). The samples with diamond structure were designed with porosities: 81%, 73%, 50% and 34%. The samples with porosity near 0 were also included (Table 2).

**Figure 2:**
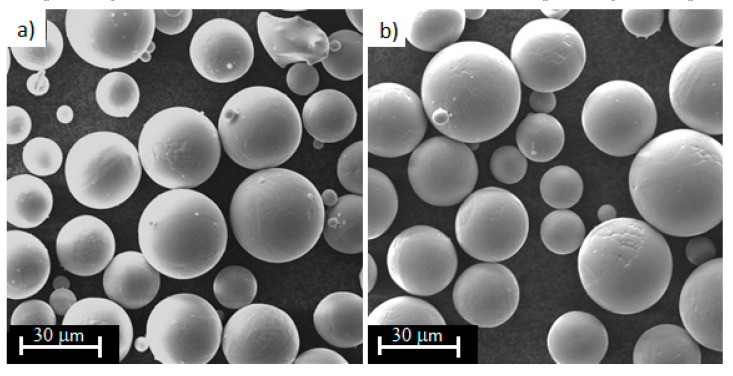
Morphology of the Ti6Al4V titanium alloy powder used during the printing process: (**a**) new powder, (**b**) reused powder (20 times).

The test samples were designed and manufactured by Medgal® Orthopeadic Implants and Instruments, which has extensive experience in the manufacture of orthopaedic implants and medical instruments. Proposed structures were made according to exact technology used to produce elements of joint endoprostheses offered by this company. The samples were printed in the ProX DMP 320 printer, whose producer is 3DSystems (Los Angeles, CA, USA). It is a device enabling industrial production of large parts. The performance of the printer is increased by the validated system, process control or power management and recycling procedure. It allows the powder to be reused (up to 20 times) without any loss of product quality. This is demonstrated in the microscopic images of the new powder and reused powder, which were done on the Phenom XL scanning electron microscope (Thermo Fisher Scientific, Waltham, MA, USA). The powder particle looks identical in both images, so reuse of the powder does not change it (Figure 2). In addition, the printer produces chemically clean and durable components, which is due to the low oxygen concentration in the working chamber during the printing process. The values of the printer parameters used in this process have been optimized by the manufacturer. The layer thickness is 60 µm, scanning speed – 400 mm/s. The printing time is between 35 min and 1 hour 40 min, depending on the porosity.

**Figure 3:**
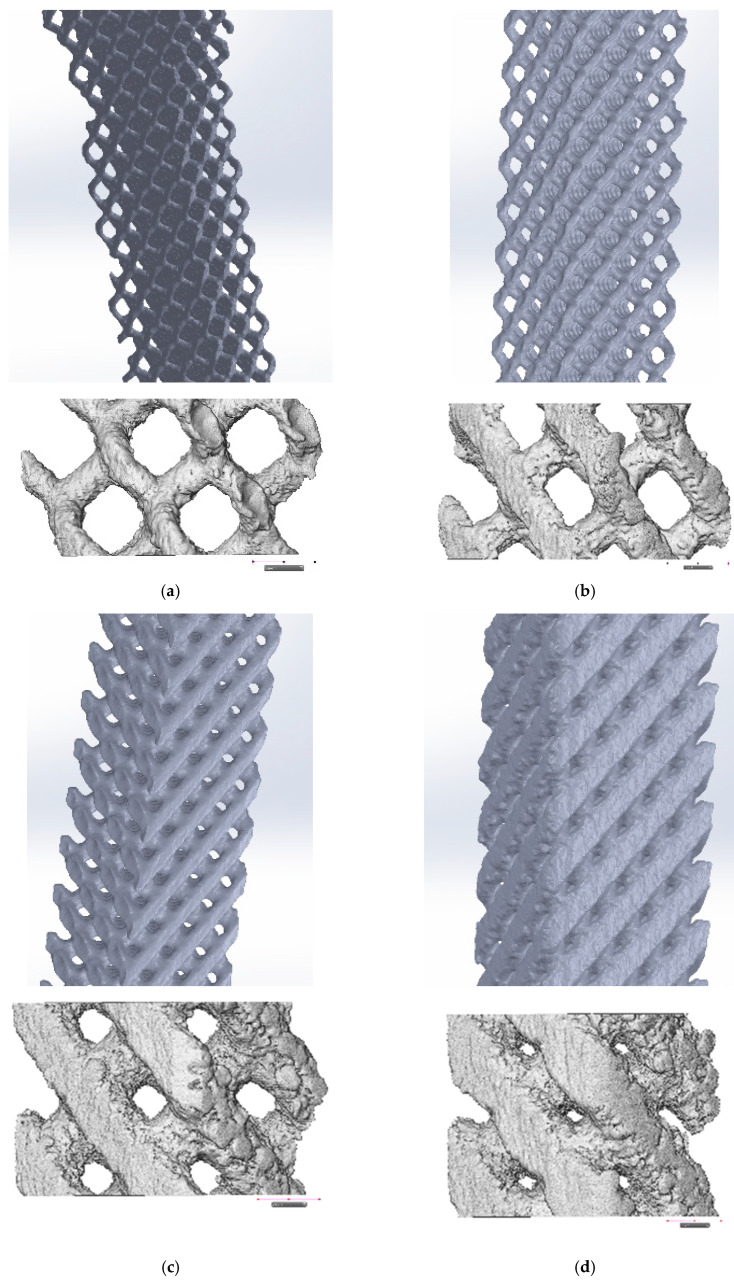
Spatial, tomographic and microtomographic images of the structure of samples of the Ti6Al4V titanium alloy with diamond structure and porosity: (**a**) 81%; (**b**) 73%; (**c**) 50%; (**d**) 34%.

**Figure 4:**
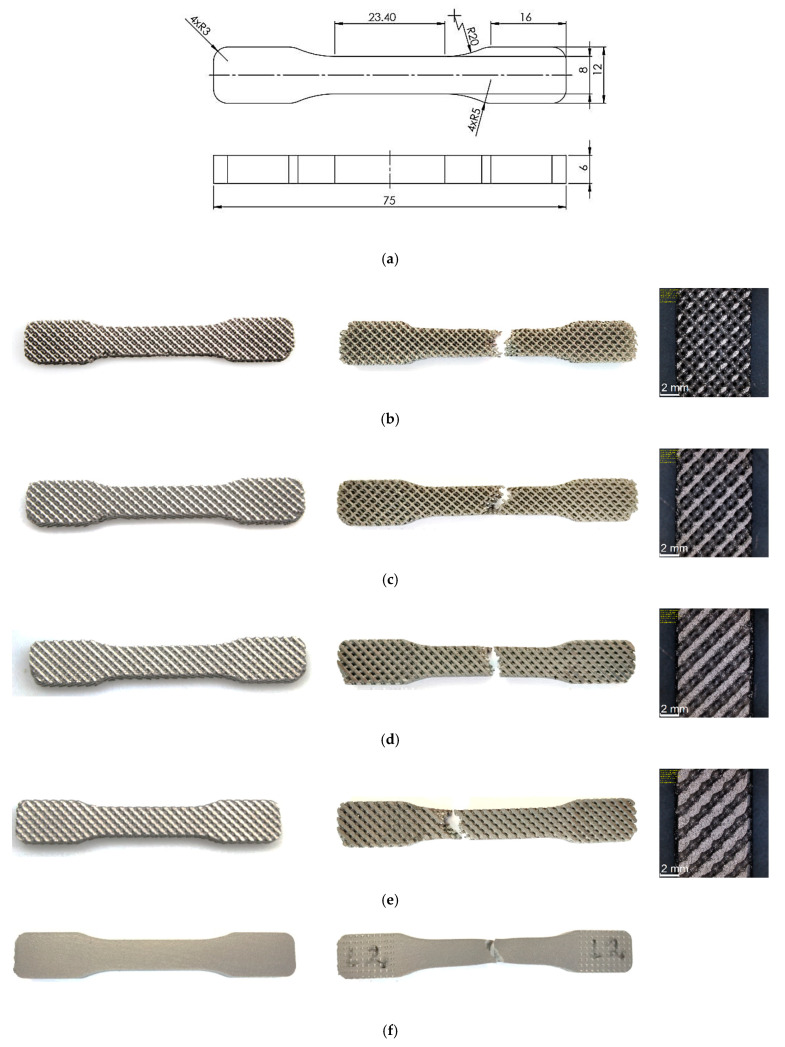
Dimensions of specimens for tensile testing (**a**) and specimens of Ti6Al4V titanium alloy printed by Laser Power Bed Fusion (LPBF) (before and after testing) with porosities: 81% (**b**); 73% (**c**); 50% (**d**); 34% (**e**) near 0 (**f**).

In the process of sample manufacturing, the most parameters were based on published research [46], except hot isostatic pressing (HIP) which was not performed. After sintering, the samples were placed in a vacuum furnace at 920 °C in order to eliminate internal stresses. Next they were cut off from the working tray. At the end the supports were removed. The samples were sand-blasted and grinded. To observe the elements of the internal structure in detail, spatial measurements were also taken on CT and SkyScan 1172 computer microtomographs by Bruker (Billerica, MA, USA) (Figure 3). In addition, the samples were weighed and measured (thickness and width). The analytical balance was used with an accuracy of 0.001 g. To measure the dimensions of the sample a micrometer with an accuracy of 0.01 mm was used. The specimens before tensile tests as well as the specimens after the test are shown in Figure 4.

The specimens were subjected to monotonic tensile tests according to ASTM standard [47]. Each test was performed on MTS 858 Mini Bionix with digital control of FlexTest SE (MTS Systems Corporation, Eden Prairie, MN, USA). Changes in the gauge length were recorded using an Instron 2620-201 extensometer with a gauge length of 25 mm and a range of ±5 mm. The load reference speed was 0.01 mm/s and the data recording frequency was 25 Hz. Tensile test was performed until total specimen break. Three repetitions were made for each sample type and the results were averaged.

To obtain Poisson ratio two extensometers were used. An Instron 2620-201 extensometer (Norwood, MA, USA) with a measuring length of 25 mm was used to determine the effective axial strain, and an MTS 632.18F-20 diameter extensometer was used to measure the effective cross-sectional strain.

## 3. Results and Discussion

**Figure 5:**
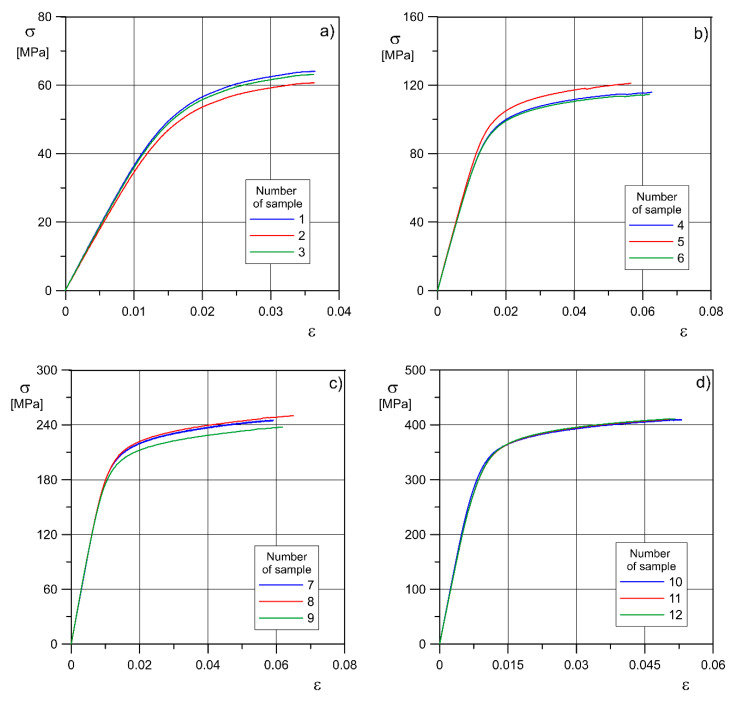

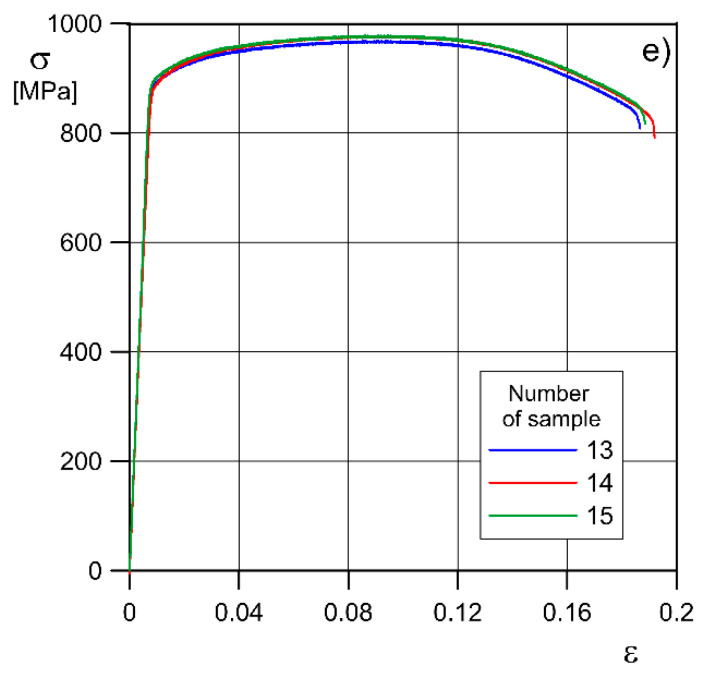
Monotonic tensile testing of Ti6Al4V titanium alloy specimens printed with LPBF method with porosities: (**a**) 81%; (**b**) 73%; (**c**) 50%; (**d**) 34%; (**e**) near 0, where: *σ*—nominal axial stress, *ε*—nominal strain (both effective).

The results of the monotonic tensile test and the most significant effective strength properties are shown in Table 3 and Figure 5. The obtained values, depending on the porosity, were compared with those of solid material.

Average values of effective strength properties of the material (Table 3) were determined, such as: axial modulus of elasticity *E*_eff_, offset yield stress σ_0.2eff_, ultimate tensile strength *σ*_ueff_ and relative elongation *A*_eff_. Let us note that the notification ‘effective’ means that all properties are related to the whole volume of porous material (like for homogeneous material). The effective value of Poisson’s ratio was also determined. These effective mechanical properties strongly depend on the density of the material (i.e., its porosity). In the case of the diamond structure with the highest density (*p* = 34%), the value of the effective axial modulus of elasticity *E*_eff_ is more than 10 times higher than in the case of the structure with the lowest density (*p* = 81%). The value of the effective tensile strength and yield strength increases about six times. As the porosity decreases, an increase in the effective relative elongation is also observed, except in the structure with 34% porosity. It is difficult to explain such behavior of sinters studying the experimental results only, therefore it is necessary to make a numerical analysis of deformation and fracture of the material structure using the finite element method and computer microtomography [48,49]. The values of effective strength properties obtained for structures with the lowest porosity are almost three times smaller than those imitating solid material. However, the essence of using porous structures is to replace damaged bone tissue. It should be remembered that the strength of bone is much lower than strength of solid titanium alloy Ti6Al4V. 

The values of the strength parameters obtained for the material produced by the LPBF method of near-zero porosity obtained from the monotonic tensile test are similar to those of the solid material given by the producer of the sample powder [45]. It proves the appropriately selected technological parameters during the LPBF process.

**Figure 6:**
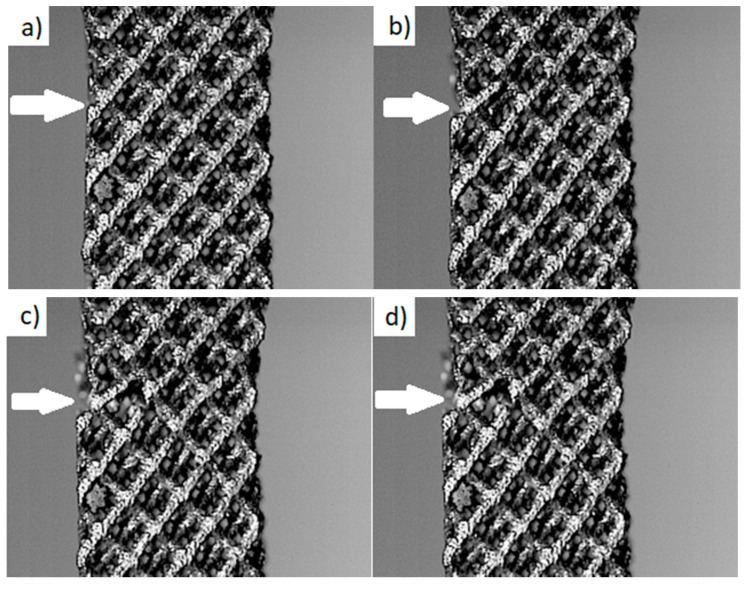

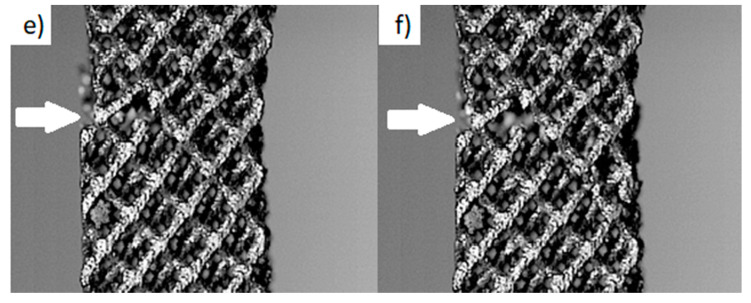
Crack initiation and propagation up to destruction in an expanded Ti6Al4V titanium alloy sample with a porosity of 81%: crack initiation at the first bar (**a**), cracking of the first bar (**b**), cracking of the next bars (**c**–**e**), rupture of the sample (**f**).

The monotonic tensile test of Ti6Al4V titanium alloy sample with a porosity of 81% was recorded with a high-speed Phantom V1610 camera. The process of initiation and propagation of a crack to final failure of the sample under the tensile force is shown in Figure 6. It should be noted that the single beams get failure one by one. The weakest beams on the edge of the sample break first. This is mainly related to the method of sample manufacturing, since the edge beams may have both an “incomplete” (unfinished) structure and the laser energy required to melt the powder particles at the edge may be slightly lower.

When analyzing the fracture surfaces of specimen after the monotonic tensile test, it can be seen that the crack planes of the individual beams are located at different angles to the tensile axis and regardless of the porous structure. The angle of fracture plane orientation ranges from 25° to 50°. The order of failure of the single beams has a large influence on the value of this angle. This shows that the failure process depends on the values of normal and shear stresses in these planes.

**Figure 7:**
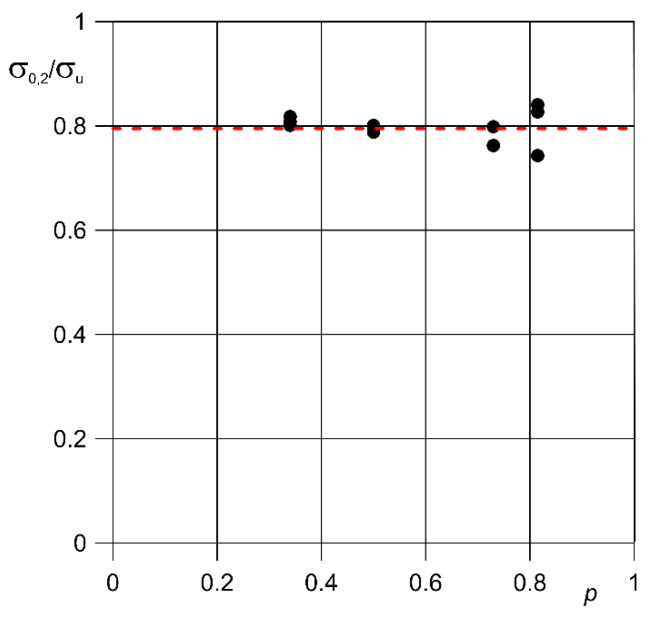
Relationship between the effective values: yield strength to tensile strength of porous samples of Ti6Al4V titanium alloy obtained by LPBF method with different porosity.

Identical technological parameters during 3D printing allow repeatable results to be obtained. This can be confirmed by analyzing the monotonic test results. This can also be confirmed by comparing the relationship of the effective yield strength σ_0.2 eff_ to the effective tensile strength σ_ueff_ (Figure 7). For all samples with a porous structure the value of this relationship was approximately 0.8 (Table 4).

Obtaining the monotonic tensile curves made it possible to determine the averaged hardening curves of the material, taking into account different degrees of structure density, by approximation of the tensile curve in the range of plastic deformations, according to the Ramberg–Osgood Equation:

(1) $$\sigma =K\varepsilon^{p^{n}}$$

where: *ε*^p^—effective axial plastic deformation, σ—effective axial stress corresponding to this deformation. The values of the coefficients *K* and *n*, determined by approximation with Equation (1) are shown in Table 5. They depend on the density of the investigated diamond structure. The values of the correlation coefficient R^2^ are also presented in this table.

Table 6 shows the values of the effective strength limit for porous material related to the effective strength limit for solid material (obtained also in LPBF technology). The coefficient of decrease of effective strength ξ (with the increase in porosity) has been introduced, namely:

(2) $$\xi =\frac{\sigma_{\mathrm{ueff}}}{\sigma_{u}\left(p=0\right)}=\frac{\sigma_{\mathrm{ueff}}}{\sigma_{u0}}.$$

This coefficient can be determined by an Equation:

(3) $$\xi ={\left(1-p\right)}^{m}$$

**Figure 8:**
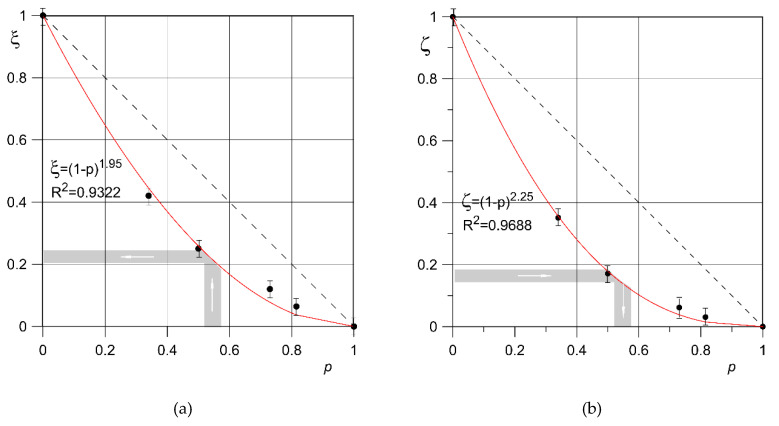
Values of *ξ* (**a**) and *ζ* (**b**) coefficients for samples of Ti6Al4V titanium alloy with diamond structure and different porosity.

As an approximation of the quotient of the effective tensile strength *σ*_ueff_ of samples from titanium sinters of porosity *p* to the tensile strength *σ*_u0_ of the sample with near-zero porosity obtained by the LPBF method, determined experimentally (Table 6). The compliance of this function with the experimental results is shown in Figure 8a.

The change in the effective axial modulus of elasticity can be described in a similar way, together with the change in material porosity, namely:

(4) $$\zeta =\frac{E_{\mathrm{eff}}}{E\left(p=0\right)}=\frac{E_{\mathrm{eff}}}{E_{0}}.$$

The ratio of effective axial modulus of elasticity of porous samples *E*_eff_ to axial modulus of elasticity of a sample with near-zero porosity *E*_0_ can be determined by an Equation (Figure 8b):

(5) $$\zeta ={\left(1-p\right)}^{k}$$

The proposed Equations (3) and (5) allow for the estimation of the usefulness of the proposed material structure, obtained by the LPBF method, for practical use, e.g., in implant engineering. The higher the values of *m* and *k* exponents, the faster the relative decrease of the effective tensile strength and the effective axial modulus of elasticity of the material with an increase in porosity. In the case of analyzed diamond structure made of titanium alloy Ti6Al4V by LPBF method, quite similar values of these exponents were obtained: *m* = 1.95 and *k* = 2.25. It shows that with relative decrease of material stiffness (desired effect) there is a slightly smaller relative decrease of strength (undesirable effect), because *m* < *k*. The greater the difference between the *k* and *m* exponents, the more relative decrease in effective modulus of elasticity is not accompanied by a comparable relative decrease in effective tensile strength of the material. Equations (1)–(3) can therefore be used both to verify structure correctness of a porous material and to design structures with defined stiffness and strength.

The range of axial modulus of elasticity, which is between 17.6 and 21.5 GPa [50], corresponds to the value of the coefficient *ζ* from 0.15 to 0.18. It is the value of Young’s modulus of bone with reference to solid titanium alloy (Figure 8b). This range of modulus of elasticity corresponds to a range of porosity from 52.5% to 56.5%. For such porosity the values of *ξ*, that is the effective tensile strength of the structure related to the tensile strength of a solid titanium alloy, can be determined (Figure 8a). In this case they range from 0.20 to 0.23, which corresponds to the effective tensile strength of the diamond structure from 194.4 to 228.4 MPa. This value should be compared with the stress value obtained during strength calculations of an implant element such as a pin of hip endoprosthesis. 

**Figure 9:**
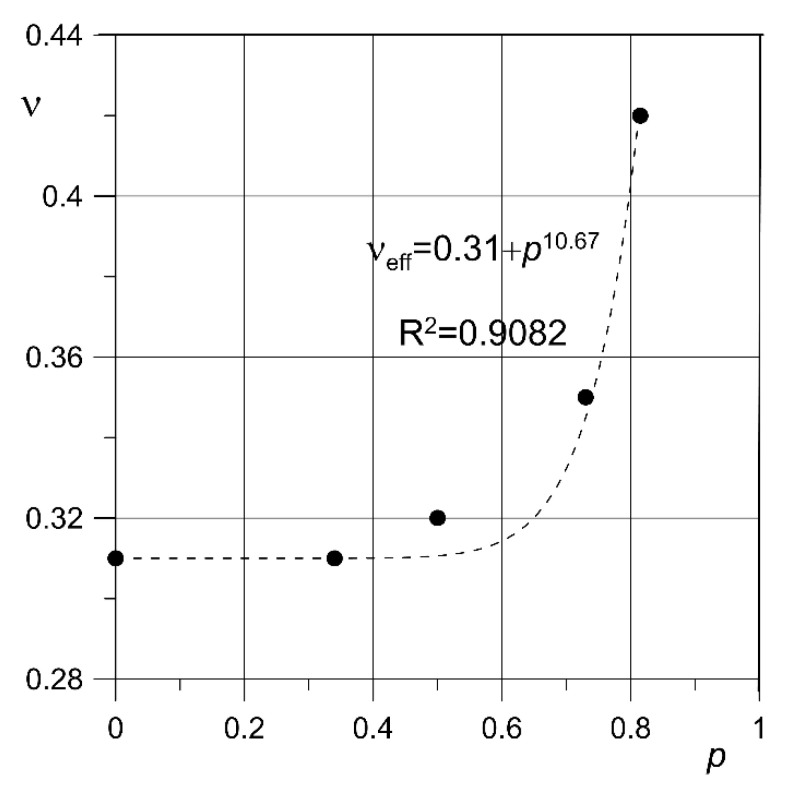
Effective Poisson’s ratio values approximated by Equation (6).

The dependence of the effective Poisson’s ratio value on the porosity of the diamond structure was also determined. The obtained values are shown in Table 3 and Figure 9. It should be noted that effective Poisson’s ratio increases with porosity, especially for its two highest values. This is due to the fact that in addition to the cross-sectional deformation of the material, there is also a change in the shape of its structure, particularly visible for porosity *p* = 73% and 81%. The obtained results can be approximated by the form dependence:

(6) $$\nu_{\mathrm{eff}}=\nu_{0}+p^{c_{\nu }}.$$

where *ν*_eff_—effective Poisson’s ratio for a material with porosity *p*, *ν*_0_—Poisson’s ratio for near-zero porosity, c*_ν_*—an exponent of constant value for a given structure, determined from experimental results. It should be added that Equation (6) can only be applied for a porosity no greater than the highest value for which the Poisson’s effective ratio was experimentally determined.

The fracture surfaces obtained from the monotonic tensile test were subjected to microscopic analysis on the Phenom XL Desktop SEM scanning electron microscope.

**Figure 10:**
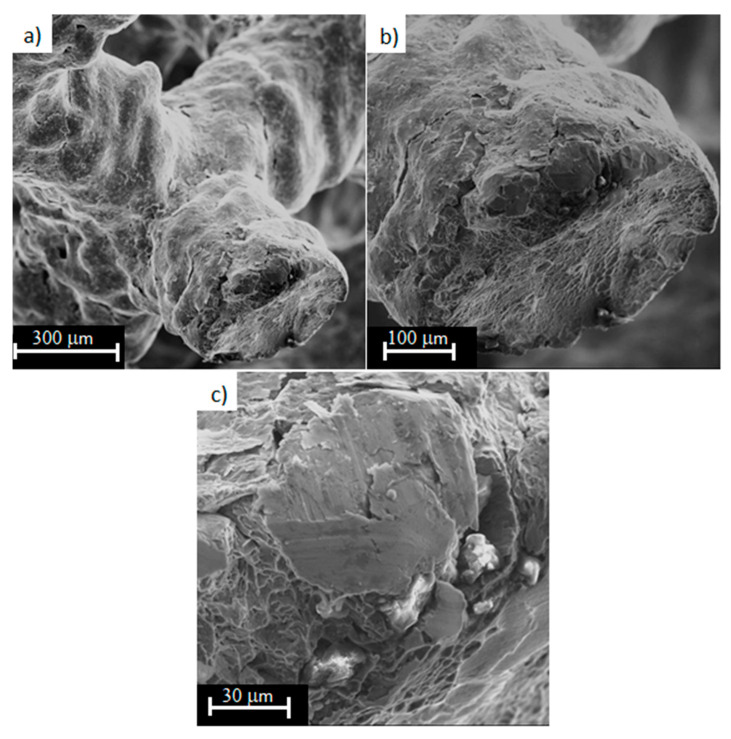
Fracture surfaces of Ti6Al4V titanium alloy with 81% porosity, subjected to a monotonic tensile test (magnification: (**a**) 250×; (**b**) 500×; (**c**) 2000×).

Figure 10 shows the areas of decohesion of the structure of the sample made of titanium alloy Ti6Al4V obtained by LPBF process with a porosity of 81%, caused by tensile force. It can be seen that the material is under a complex stress state (tensile and shear) during tension. This is due to the fact that the beams forming the diamond structure of the sample are arranged at an angle of about 45° to the tensile direction. At the fracture surfaces, the areas of ductile fracture are dominating (Figure 10b). Shear areas were also observed (Figure 10c). Stress concentration occurs especially in the area of structure defects (e.g., notches), which results in significant deformation and subsequent fracture. In Figure 10a,b, cracks in a single beam can be observed in several places at once. This may be due to the action of technological microcracks (after all, the side surface of the barrel is not smooth, see Figure 3), which caused the initiation of these cracks.

**Figure 11:**
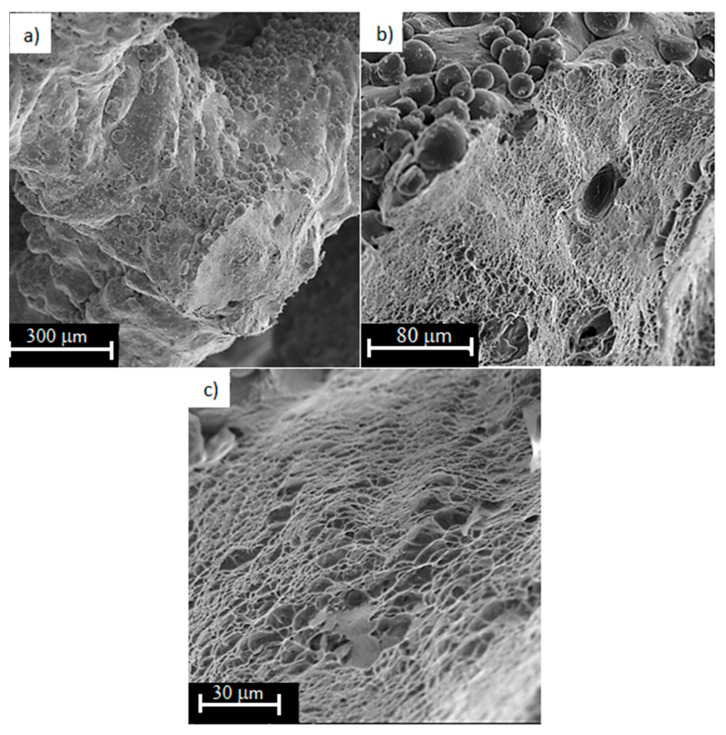
Fracture surfaces of Ti6Al4V titanium alloy with 73% porosity, subjected to a monotonic tensile test (magnification: (**a**) 250×; (**b**) 1000×; (**c**) 2000×).

Analyzing the fracture surfaces of Ti6Al4V samples with 73% porosity, the surface of the beads shows characteristic balls of different sizes (Figure 11a,b). Possibly they could be un-melted and thus unbound powder particles, forming the “balling effect”. This effect occurred during the printing process and its intensity progresses with the reduction of energy density [33,51,52,53]. At the fracture surfaces, the areas of ductile fracture with visible defects in the form of gas pores are noted. These areas were created by insufficient binding of the powder. The pores can reach about 30–40 µm for the analyzed area. This is a typical defect in the metallic structure and occurs on the laser treated surface, becoming an obstacle to the application of another new layer of powder. Some studies have shown that the size of these pores reach almost 200 µm [8]. 

**Figure 12:**
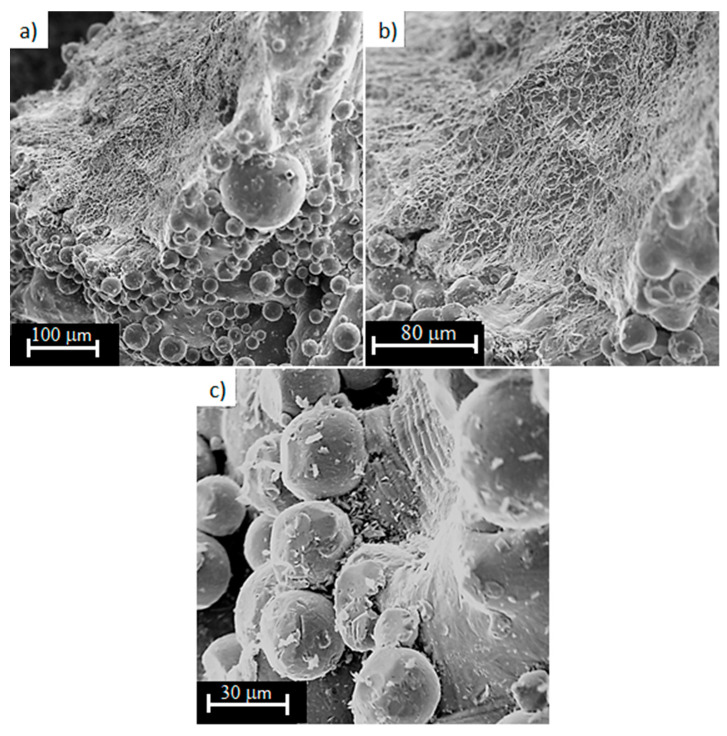
Fracture surfaces of Ti6Al4V titanium alloy with 50% porosity, subjected to a monotonic tensile test (magnification: (**a**) 500×; (**b**) 1000×; (**c**) 2000×).

**Figure 13:**
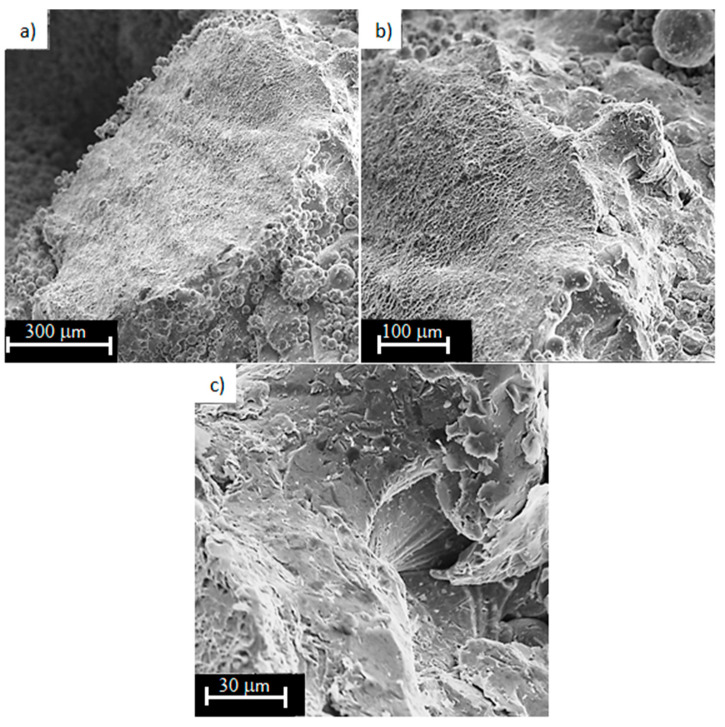
Fracture surfaces of Ti6Al4V titanium alloy with 34% porosity, subjected to a monotonic tensile test (magnification: (**a**) 250×; (**b**) 500×; (**c**) 2000×).

For the analysis of structures with a porosity of 50% and 34%, balls with a diameter of up to 100 µm are observed (Figure 12a), although they may be un-melted or partially melted powder particles [53,54,55]. On the side surface of the fracture surfaces (Figure 12c), slip bands in single grains and cracks in this area can be observed. On samples with a porosity of 34%, there are less gaseous pores causing structural defects (Figure 13a). For porosity of 50% and less, it should be noted that the majority of the fracture surfaces have a ductile character. However, in this case the surface of it is smoother. This may be due to the greater homogeneity of the diamond structure beams material.

**Figure 14:**
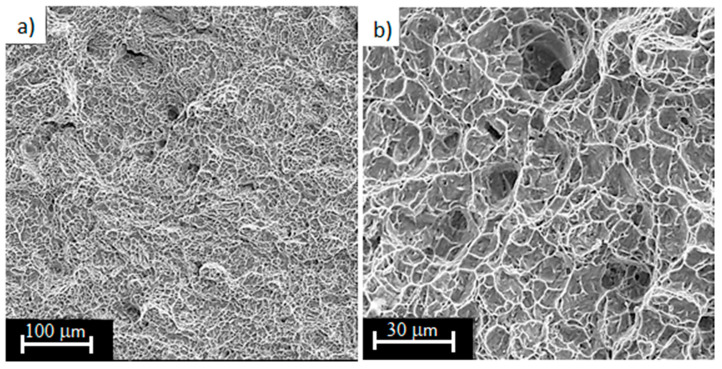
Fracture surfaces of Ti6Al4V titanium alloy with near-zero porosity, subjected to a monotonic tensile test (magnification: (**a**) 500×; (**b**) 2000×).

Samples of Ti6Al4V titanium alloy with near-zero porosity were also observed (Figure 14). From the nature of the fracture surfaces it can be concluded that the structure produced by LPBF method is not homogeneous. This can be interpreted as a result of local hardening of the material during laser processing, which could provide unequal energy to individual areas of the created structure, as well as cracks at the boundaries of the separation in the material and formation of porous structure.

## 4. Conclusions

This paper presents the results of experimental research on the strength properties of porous structures with different degrees of density produced from titanium alloy Ti6Al4V by LPBF. Monotonic tensile tests were carried out to determine the effective values of such parameters as: axial modulus of elasticity *E*_eff_, ultimate tensile strength *σ*_ueff_, offset yield strength *σ*_0.2eff_ and relative elongation *A*_eff_ for samples made of titanium alloys with diamond structure taking into account different porosity. For all parameters a significant decrease in their values was obtained with increasing porosity. Microscopic analysis of the structures using scanning microscopy and microtomography is also presented. The paper also proposes semi-empirical relationships that can be easily used to estimate the strength and stiffness of porous material obtained by 3D printing in these conditions. This can be the starting point for research on other materials and structures.

Porous structures allow the use of metallic materials as replacements for joint endoprostheses. This is possible mainly as a result of a reduction in stiffness. At the same time, however, there is a decrease in the value of strength properties. Therefore, the optimal solution is to use such a degree of density of a porous structure that would combine a stiffness similar to that of bone tissue while simultaneously obtaining strength parameters which would be sufficient for the applied loads.

Relationships, based on the results of experiment, were proposed to determine the effect of porosity of the diamond structure on the strength and stiffness of the studied material. They enable both the assessment of the usefulness of the analyzed structure for engineering applications, in particular in the field of implant engineering, and the proper selection of the structure, taking into account its stiffness and strength. The proposed relations concern the diamond structure obtained by the LPBF method. These dependencies must be verified by new experimental tests when using a titanium alloy Ti6Al4V with a different structure or made by different technology.

In the next stage of research the numerical modelling of deformation processes and the failure of the structure of porous sinters produced from titanium alloy Ti6Al4V by LPBF method is planned. The finite element method and images of the actual structure of materials obtained by computer microtomography will be used for this purpose. This approach was previously used in the strength tests of porous sinters of austenitic steel 316L with different degrees of structure density, obtained by powder metallurgy [56,57]. Fatigue tests of porous materials of diamond structure obtained by LBPF method are also planned. It should be remembered that the appropriate strength, fatigue life and stiffness determine the non-failure operation of the implant obtained with the additive method.

## Figures and Tables

**Table 1:** The chemical composition of the powder used to samples production [45].

LaserForm^TM^Ti Gr 23 (ASTM E8M)								
Al	C	Fe	H	N	O	V	Y	Others Together
5.50–6.50%	≤0.08%	≤0.25%	≤0.12%	≤0.03%	≤0.13%	3.50–4.50%	≤0.005%	≤0.40%

**Table 2:** Types of samples of Ti6Al4V titanium alloy obtained by the LPBF method used in the tests.

Type of Sample	Density (%)	Porosity (%)	Beam Thickness (mm)	Weight (g)
Type 1	19	81	0.49	4.13
Type 2	27	73	0.60	6.11
Type 3	50	50	0.70	10.77
Type 4	66	34	1.20	14.48
Type 5	100	near 0	–	19.85

**Table 3:** Effective strength properties of Ti6Al4V sintered titanium alloy of different porosities produced by the LPBF method compared with the values for solid material specified by the producer.

Sample No.	*p* (%)	*E* (GPa)		*ν*	*σ*_u_ (MPa)		*σ*_0.2_ (MPa)		*A*	
1	81	3.8	3.7	0.42	64.0	62.6	47.6	50.3	0.037	0.036
2		3.6			60.7		51.0		0.036	
3		3.7			63.1		52.2		0.036	
4	73	7.3	7.4	0.35	115.9	117.2	88.4	90.8	0.063	0.061
5		7.3			121.1		96.7		0.057	
6		7.5			114.7		87.4		0.062	
7	50	19.9	20.4	0.32	245.1	244.2	196.3	193.5	0.065	0.064
8		20.6			249.9		196.9		0.062	
9		20.6			237.5		187.3		0.065	
10	34	41.3	41.8	0.31	409.0	410.0	334.5	331.7	0.053	0.052
11		42.8			410.1		331.5		0.051	
12		41.3			410.9		329.1		0.052	
13	-	120.1	-	0.31	969.0	-	899.5	-	0.187	-
14	near 0	116.5	118.7		977.6	975.1	895.9	898.8	0.192	0.189
15	-	119.5	-		978.7	-	900.9	-	0.188	-
Solid material		105–120		0.31–0.37	940 ±50 MPa * 1080 ± 100 MPa **		850 ± 100MPa * 1000 ±100MPa **		0.15 ± 0.05 * 0.11 ± 0.03 **	

* After hot isostatic pressing [45], ** After annealing [45].

**Table 4:** Averaged values of the effective yield strength to tensile strength of Ti6Al4V titanium alloy samples of different porosity, obtained by LPBF method.

*p*	*σ*_0.2eff_ (MPa)	*σ*_ueff_ (MPa)	*σ*_0.2eff_/*σ*_ueff_
0.34	331.7	409.9	0.81
0.5	193.5	244.2	0.79
0.73	90.8	117.3	0.77
0.81	50.3	62.6	0.80

**Table 5:** Average values of coefficients and exponents of the monotonic, effective hardening curve of Ti6Al4V titanium alloy diamond structure with different porosity.

Porosity (%)	*K* (MPa)	*n*	*R* ^2^
81	110.66	0.17	0.9961
73	163.98	0.12	0.9914
50	317.69	0.09	0.9980
34	519.64	0.08	0.9952

**Table 6:** Values of the coefficients *ξ* and *ζ* for samples of Ti6Al4V titanium alloy with different porosity obtained by the LPBF method during the tensile test.

*p*	*σ*_u_ (MPa)	ξ *σ*_u_/*σ*_u0_	*E* (GPa)	ζ *E/E*_0_
near 0	975.09	1	118.7	1
0.34	409.98	0.44	41.8	0.38
0.5	244.15	0.26	20.4	0.20
0.73	117.31	0.08	7.4	0.05
0.81	62.63	0.04	3.7	0.02
